# Quantitative T2-Mapping and T2^⁎^-Mapping Evaluation of Changes in Cartilage Matrix after Acute Anterior Cruciate Ligament Rupture and the Correlation between the Results of Both Methods

**DOI:** 10.1155/2018/7985672

**Published:** 2018-05-17

**Authors:** Hongyue Tao, Yang Qiao, Yiwen Hu, Yuxue Xie, Rong Lu, Xu Yan, Shuang Chen

**Affiliations:** ^1^Department of Radiology, Affiliated Huashan Hospital of Fudan University, Shanghai, China; ^2^Shanghai Proton and Heavy Ion Center, Shanghai, China; ^3^MR Collaboration NE Asia, Siemens Healthcare, Shanghai, China

## Abstract

**Objectives:**

To quantitatively assess changes in cartilage matrix after acute anterior cruciate ligament (ACL) rupture using T2- and T2^⁎^-mapping and analyze the correlation between the results of both methods.

**Methods:**

Twenty-three patients and 23 healthy controls were enrolled and underwent quantitative MRI examination. The knee cartilage was segmented into six compartments, including lateral femur (LF), lateral tibia (LT), medial femur (MF), medial tibia (MT), trochlea (Tr), and patella (Pa). T2 and T2^⁎^ values were measured in full-thickness as well as superficial and deep layers of each cartilage compartment. Differences of T2 and T2^⁎^ values between patients and controls were compared using unpaired Student's *t*-test, and the correlation between their reciprocals was analyzed using Pearson's correlation coefficient.

**Results:**

ACL-ruptured patients showed higher T2 and T2^⁎^ values in full-thickness and superficial layers of medial and lateral tibiofemoral joint. Meanwhile, patients exhibited higher T2^⁎^ values in deep layers of lateral tibiofemoral joint. The elevated percentages of T2 and T2^⁎^ value in superficial LT were most significant (20.738%, 17.525%). The reciprocal of T2^⁎^ value was correlated with that of T2 value (*r* = 0.886, *P* < 0.001).

**Conclusion:**

The early degeneration could occur in various knee cartilage compartments after acute ACL rupture, especially in the superficial layer of LT. T2^⁎^-mapping might be more sensitive in detecting deep layer of cartilage than T2-mapping.

## 1. Introduction

The anterior cruciate ligament (ACL) is the primary constraint to the anterior translation and internal rotation of the tibia and acts as an important stabilizer of the knee. ACL sprain or tear constitutes one of the most common types of knee injuries during sports [1]. Acute ACL rupture increases the instability and alters the biomechanical properties of the knee joint [2], which can lead to pathological changes in cartilage matrix and eventually cartilage degeneration over time [3]. Due to recurrent instability, acute ACL rupture has been suggested as a high-risk factor for osteoarthritis (OA) onset and development [4]. Therefore, quantitative assessment of early cartilage degeneration in knees with ACL injury is crucial for detecting cartilage degeneration at a subclinical stage when changes are potentially still reversible and for guiding future treatment modalities [5, 6].

Magnetic resonance imaging (MRI) has been demonstrated to be a useful and noninvasive imaging tool for the assessment of pathological alterations in cartilage morphology [7, 8]. In the case of OA, however, it has been suggested that early degeneration can occur in the cartilage matrix prior to morphological changes, such as the loss of cartilage thickness, becoming detectable during standard MRI scans [9, 10]. In this regard, the multi-echo T2-mapping can be employed to assess the concentration, orientation, and integrity of collagen and water content in cartilage matrix [11, 12] and has been suggested as a sensitive technique for early detection of cartilage degeneration [13].

Compared to T2-mapping, quantitative T2^*∗*^-mapping has demonstrated a number of benefits including short scan time and high resolution. Recent studies on the application of quantitative T2^*∗*^-mapping have shown promising results for the evaluation of cartilage matrix [14]. T2^*∗*^ is defined as the observed rate constant for the decay of transverse magnetization that includes both T2 relaxation and coherent dephasing effects as a result of local magnetic field inhomogeneity. The relationship between T2^*∗*^ and T2 follows the equation 1/T2^*∗*^ = 1/T2 + *γ*ΔB [15]. Thus, assuming the uniformity and stability of the applied static magnetic field (*B*_0_), T2^*∗*^ would depend on both T2 and local susceptibility-induced magnetic fields. In the context of cartilage imaging, it has been suggested that these fields often play a significant role at the bone-cartilage interface and within the fundamental cartilage microarchitecture, which could translate into changes in T2^*∗*^ readings [15]. This thus implies that T2^*∗*^-mapping might be a more sensitive imaging strategy for identifying cartilage degeneration of calcified layers [15]. Indeed, T2^*∗*^-mapping was found to offer the advantages of shorter scan time and compatibility with 3D acquisition over T2-mapping in the visualization of articular cartilage [16]. The method has also exhibited satisfactory sensitivity to cartilage matrix changes in ankle osteochondrosis dissecans and hip femoroacetabular impingement [17, 18].

To date, very few studies have compared T2^*∗*^- and T2-mapping techniques in the assessment of cartilage matrix changes after acute ACL rupture. In addition, little is known regarding the detailed topographic degeneration of knee cartilage after acute ACL rupture. To address these needs, the current study applies T2- and T2^*∗*^-mapping to the visualization of cartilage matrix in patients with acute ACL rupture and healthy controls and analyzed the correlation of the results of two methods. We hypothesized that patients with acute ACL rupture would show an elevated T2 and T2^*∗*^ value at various compartments of knee cartilage when compared with healthy subjects. Additionally, the reciprocal of T2^*∗*^ value might closely correlate with that of T2 value.

## 2. Materials and Methods

### 2.1. Patient Population

The study was performed according to guidelines approved by the health sciences institutional review board of our hospital. Written consent was obtained from all participants prior to the experiments. The selection criteria for patients included clear evidence of unilateral ACL rupture as shown by MRI or clinical examination and confirmed by arthroscopy [19], no prior injury in the contralateral knee, an interval of 2 to 6 months between the rupture and the MRI scan, age between 18 and 50 at the time of the rupture, and a body mass index (BMI) below 25 kg/m^2^. Exclusion criteria comprised knee OA, inflammatory arthritis, knee trauma, posterior cruciate ligament tear, medial or lateral collateral ligament injury, and serious meniscus corner injury that required surgery. Patients who had previously received knee surgery were also eliminated from consideration. For the selection of healthy volunteers, who served as controls, candidates were excluded if they had a history of disease, injury, or surgical intervention in the knees, or if their MRI scans revealed any of the abovementioned knee abnormalities. The eligible healthy participants were matched to the patients according to sex, age, and BMI. One knee of each healthy subject was randomly selected for MRI examination. Thirty patients were diagnosed by standard MRI as having ACL rupture without tear of collateral or posterior cruciate ligament, among whom 27 subsequently were confirmed by arthroscopy during reconstruction surgery. Among the 27 patients, four were excluded due to the detection of serious meniscal injury that warranted surgical treatment. On the other hand, three of the 26 asymptomatic healthy volunteers were also excluded, including one with ACL abnormality and another two with meniscal injury showed by MRI examination. After the prescreening, the final study cohort consisted of an ACL rupture group of 23 patients and a control group of 23 healthy adults, with no significant difference in sex, age, or BMI between the two. The mean interval time from rupture to MRI scan was 3.5 months, whereas the mean interval period between MRI examination and surgery was 2.5 months. Participants' demographic data was shown in Table 1.

### 2.2. Imaging Acquisition

All MRI scans were performed on a 3T MRI unit (Verio, Siemens, Germany) with a gradient strength of 40 mT/m an 8-channel phase array knee coil. Imaging sequences included fat-saturated proton-density weighted imaging (PD-FS), three-dimension double echo steady state sequence (3D-DESS), and quantitative MRI T2- and T2^*∗*^-mapping. The MRI parameters were optimized in order to achieve the highest signal noise ratio (SNR) and image quality. T2- and T2^*∗*^-mapping were conducted using similar imaging parameters to enable comparison. The knees were positioned in parallel to the direction of *B*_0_ [20]. Each participant was required to rest for 30 min prior to the knee scan to ensure that the cartilage is at resting condition. The entire ACL and the meniscus were imaged by oblique sagittal and midsagittal PD-FS, respectively, with the following parameters: TR/TE = 3000/33 ms, field of view (FOV) = 150 × 150 mm, slice thickness = 4 mm, matrix = 320 × 240, pixel size = 0.6 × 0.5 × 4.0 mm, number of excitations = 1, flip angle = 150 degrees, bandwidth = 240 Hz/pixel, and scan time = 2:32 min [21]. The morphology of cartilage was evaluated by sagittal 3D-DESS with TR/TE = 14.1/5 ms, FOV = 150 × 150 mm, slice thickness = 0.6 mm, matrix = 256× 238, pixel size = 0.6 × 0.6 × 0.6 mm, number of excitations = 1, flip angle = 25 degrees, bandwidth = 250 Hz/pixel, and scan time = 5:58 min.

Quantitative assessment of knee cartilage was performed in the sagittal plane using both T2-mapping (TR/TE = 1523/13.8, 27.6, 41.4, 55.2, 69.0 ms, FOV read = 160 mm, FOV phase = 100.0%, phase resolution = 100%, slice thickness = 3 mm, slices = 22, pixel size = 0.4 × 0.4 × 3.0 mm, Dist factor = 20%, base resolution = 384, phase resolution = 100%, matrix = 384 × 384, number of excitations = 1, flip angle of excitation pulse = 90 degrees, flip angle of refocusing pulse = 180 degrees, bandwidth = 228 Hz/pixel, and scan time = 5:15 min) and T2^*∗*^-mapping (TR/TE = 809/4.36, 11.9, 19.44, 26.98, 34.52 ms, FOV read = 160 mm, FOV phase = 100.0%, phase resolution = 100%, slice thickness = 3 mm, slice resolution = 73%, pixel size = 0.4 × 0.4 × 3.0 mm, slice per slab = 44, Dist factor = 20%, baes resolution = 384, phase resolution = 100%, matrix = 384 × 384, number of excitations = 1, flip angle = 60 degrees, bandwidth = 260 Hz/pixel, and scan time = 2:47 min). T2 and T2^*∗*^ values were obtained using a pixel-wise, monoexponential nonnegative least squares (NNLS) fit analysis.

**Figure 1:**
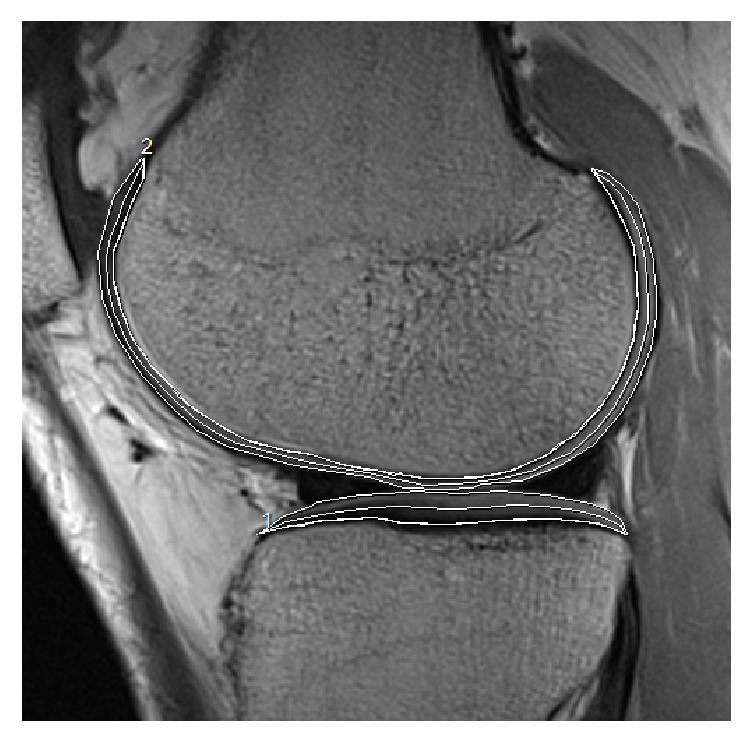
“1” and “2” showed the outlines of the lateral tibia (LT) and lateral femur (LF) cartilage compartment, respectively. LT and LF were further segmented into two equal layers (deep layer and superficial layer) when T2 and T2^*∗*^ relaxation times values were measured.

The knee cartilage was partitioned into six compartments: lateral femur (LF), lateral tibia (LT), medial femur (MF), medial tibia (MT), trochlea (Tr), and patella (Pa). In addition, LF, MF, LT, and MT were each further segmented semiautomatically by in-house software into two equal layers, the superficial layer and the deep layer (Figure 1). T2 and T2^*∗*^ relaxation times were measured from the images of each cartilage compartment and then averaged to obtain the mean T2 and T2^*∗*^ values, respectively. Subsequently, colored T2 and T2^*∗*^ maps were generated using NUMARIS/4 and Syngo MR B17 (Verio, Siemens, Germany), which could reflect the detailed topographic distribution of T2 and T2^*∗*^ values.

In 10 randomized selected patients and controls, each T2 and T2^*∗*^ measurement was performed by a main observer and repeated by another observer in order to assess the interobserver reliability. The main observer reanalyzed these subjects after 2 weeks in a randomized order to evaluate intraobserver reproducibility. In both cases, interclass correlation coefficients (ICC) were calculated and graded as follows: poor (<0.4), marginal (0.4–0.75), and good (>0.75) [22].

### 2.3. Data Analysis

Statistical analysis was performed using SPSS version 18.0 software and Microsoft Excel. Means and standard deviations (SD) of T2 and T2^*∗*^ values were calculated for each cartilage compartment. Comparison of two groups was conducted using unpaired Student's *t*-test. Pearson's correlation coefficient was used to evaluate correlation between the reciprocals of T2 and T2^*∗*^ values. *P* < 0.05 was considered statistically significant.

## 3. Results

**Figure 2:**
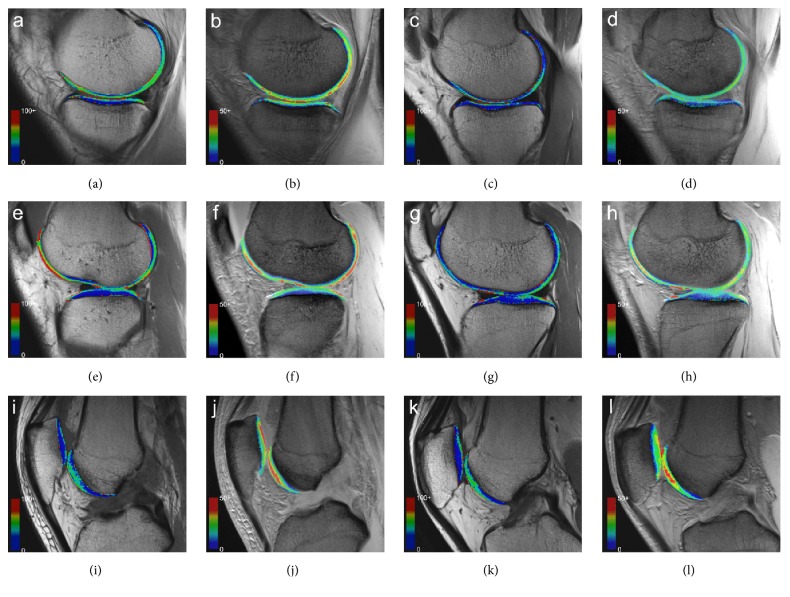
The T2-mapping (a, e, i) and T2^*∗*^-mapping images (b, f, j) of a 34-year-old patient in ACL rupture group and the T2-mapping (c, g, k) and T2^*∗*^-mapping images (d, h, i) of a 36-year-old patient in control group: according to the color bar, in the medial tibiofemoral joint (a–d), the T2 and T2^*∗*^ values in MT and MF of ACL-ruptured patient were higher than in healthy subject. In the lateral tibiofemoral joint (e–h), the patient with ACL rupture had a higher T2 and T2^*∗*^ value in LT and LF compared to the healthy subject. In the patellofemoral joint (i–l), no significant differences of T2 or T2^*∗*^ value in Pa and Tr were found between the two patients.

**Figure 3:**
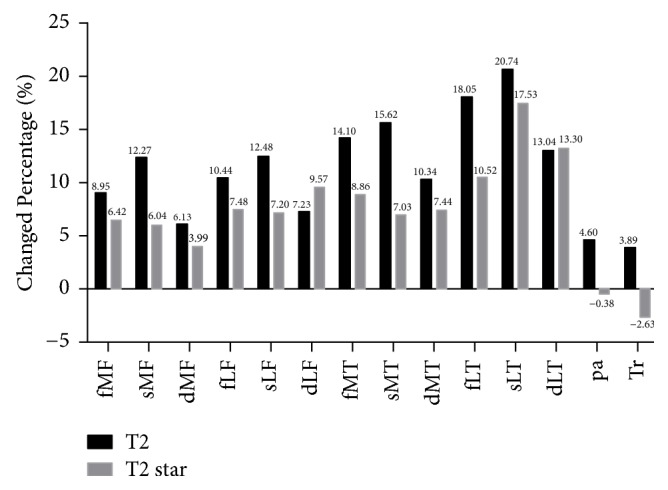
Percentage change of T2 and T2^*∗*^ value of each cartilage compartment in ACL rupture group compared to control group.

The ICC indexes of interobserver reliability and intraobserver reproducibility of T2 and T2^*∗*^ measurements were above 0.75 for all cartilage compartments in both groups. The mean T2 and T2^*∗*^ values calculated for all cartilage compartments in both groups were summarized in Table 2. Colored scaled T2-, T2^*∗*^ maps were showed in Figure 2. For the medial tibiofemoral joint, the acute ACL rupture group showed significantly higher mean T2 and T2^*∗*^ values than the control group in the full-thickness and superficial layers of MT and MF (*P* < 0.05). However, no significant statistical difference in T2 or T2^*∗*^ values was observed between the two groups in the deep layers of MT and MF (*P* > 0.05). For the lateral tibiofemoral joint, significant increases of T2 and T2^*∗*^ values were detected in the full-thickness and superficial layers of LT and LF of the patients compared to the healthy volunteers (*P* < 0.05). It is particularly noteworthy that significantly elevated T2^*∗*^ values were also registered in the deep layers of LT and LF of the patients compared to the controls (*P* < 0.05), whereas no statistically significant augmentation of T2 values was found in the same regions between the two groups (*P* > 0.05). Meanwhile, no statistically significant differences in T2 or T2^*∗*^ values were identified in the Pa or Tr compartment of the patellofemoral joint between ACL rupture patients and the controls (*P* > 0.05). The percentage increases of T2 and T2^*∗*^ values in all cartilage compartments of acute ACL rupture group over those of the control group were calculated and summarized in Figure 3. It could be seen that the elevated percentages of T2 and T2^*∗*^ value in superficial LT were most significant (20.738%, 17.525%), while those in Pa and Tr showed the smallest change (Pa: 4.598%, −0.380%; Tr: 3.886%, −2.630%).

**Figure 4:**
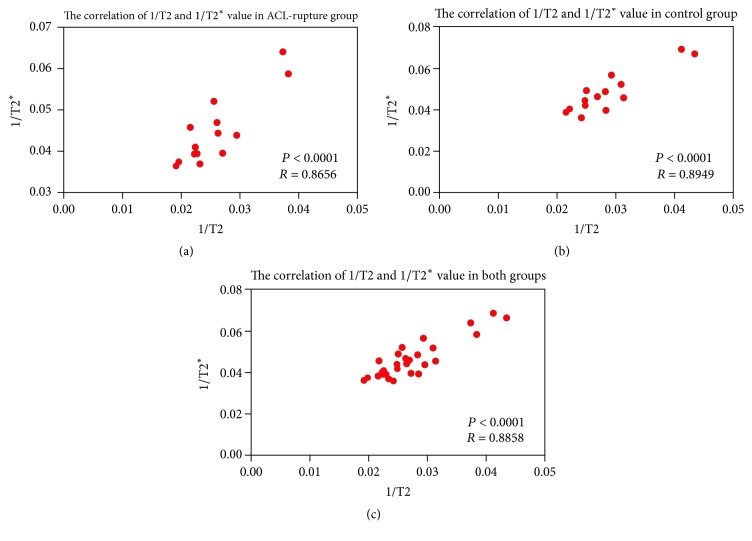
Pearson's correlation coefficient analysis of reciprocals of T2 and T2^*∗*^ in ACL rupture group (a), control group (b), and both groups (c). The reciprocal of T2^*∗*^ value was positively correlated with that of T2 value (Pearson's correlation coefficient *r* = 0.866, 0.895, and 0.886, resp., *P* < 0.001).

The reciprocals of T2 and T2^*∗*^ values of all cartilage compartments were shown to be positively correlated with each other for the acute ACL rupture group (*r* = 0.866, *P* < 0.001), the control group (*r* = 0.895, *P* < 0.001), and both groups (*r* = 0.886, *P* < 0.001), which were showed in Figure 4.

## 4. Discussion

In this study, we compared subdefined cartilage compartments in patients with acute ACL rupture to those in healthy individuals using quantitative T2- and T2^*∗*^-mapping. The acute ACL rupture group exhibited significantly higher T2 and T2^*∗*^ values than the control group in full-thickness and superficial layers of cartilage in medial and lateral tibiofemoral joint, which suggested that the early degeneration can occur in cartilage matrix in these compartments before the morphological changes occur. These results were in general agreement with several previous studies that observed higher T2 and/or T2^*∗*^ values in patients with OA compared to healthy individuals [6, 13, 18]. In contrast, no statistically significant differences in T2 or T2^*∗*^ values were found between the two groups in either Pa or Tr cartilage compartment, which means early cartilage degeneration may not occur in Pa and Tr after acute ACL rupture. As previously reported, a higher change in T2 value and T2^*∗*^ value of cartilage suggests a higher risk of OA [16–18] or even has been linked to early OA changes of the knee [13]. Therefore, an acute ACL rupture indicates the beginning of an early stage of cartilage degeneration for the tibiofemoral joint.

The observation that T2 and T2^*∗*^ differences between the two groups were generally more pronounced in the cartilage of tibial plateau than in that of femoral condyle suggested that the tibial cartilage could be more susceptible to early degeneration of cartilage matrix following acute ACL rupture. Moreover, the cartilage of LT exhibited the greatest T2 and T2^*∗*^ increases. This finding is echoed by several previously published studies. For example, Nishimori et al. [23] reported a significant association between articular cartilage damage in the posterior LT with acute ACL injury. Based on the experimental results, the authors argued that sustained injuries in this region could contribute to the eventual development of OA. Using T1*ρ* mapping, Bolbos et al. [24] suggested that ACL tear-induced pathological aberrations could occur in LT at an early stage. Similarly, Li et al. [25] revealed higher T1*ρ* values in the posterolateral tibial cartilage of patients with ACL injuries compared to healthy individuals. In addition to the above reports, our study further found that the superficial layer of LT has the highest increased T2 and T2^*∗*^ values compared with other cartilage compartments. The result showed that the superficial layer of LT may be more susceptible to reflect the ultra-early cartilage matrix degeneration after acute ACL rupture and it may play an important role in the onset and progression of OA after ACL rupture. However, it is noteworthy that a recent arthroscopic study conducted by Spindler et al. on a cohort of 54 ACL-ruptured patients found considerably more instances of cartilage lesion in the LF condyle than in the LT plateau [26]. We speculated that this could be due to the fact that the LF compartment of the knee is more readily accessible to arthroscopic inspection than LT. Taken together, our findings suggested that quantitative MRI with T2 and T2^*∗*^-mapping could complement arthroscopy in detecting pathological alterations of knee cartilage matrix, especially those occurring in the LT plateau.

In our current clinical study, the reciprocals of T2 and T2^*∗*^ values in all cartilage compartments showed a satisfactory correlation for the ACL rupture group, the control group, and for both groups combined, which responded to the physical equation 1/T2^*∗*^ = 1/T2 + *γ*ΔB. In addition, both mapping methods showed similar statistical differences between the two groups in the full-thickness and superficial layers of the whole tibiofemoral joint. Consistent results were described by Welsch et al. in their evaluation of cartilage repair in knee and by Marik et al. on osteochondrosis dissecans in talocrural joint [17, 27]. However, the two methods diverged when it came to the deep layers of lateral tibiofemoral joint, where T2^*∗*^ values between the ACL rupture group and the control group exhibited statistically significant differences not observed in T2-mapping. T2 value reflects the concentrations and orientations of collagen and the content of water in cartilage matrix, whereas T2^*∗*^ is influenced by T2 and additional factors related to the macromolecular content of cartilage based on microsusceptibility effects [16, 18, 27]. The degeneration that occurs in the calcified layer of cartilage or irregularly organized collagen in the deep radial layer could lead to variations in local susceptibility that can be detected by T2^*∗*^-mapping. Therefore, T2^*∗*^-mapping could demonstrate greater sensitivity in identifying cartilage degeneration in deep layer compared to T2-mapping. However, T2^*∗*^-mapping is also more sensitive to susceptibility artifacts, which might limit its use in patients wearing metallic joint implants [15]. In general, we believe that T2^*∗*^-mapping can serve as a useful addition to the current imaging toolbox for evaluating articular cartilage in patients with acute ACL rupture. Further studies will be needed to fully validate and optimize this technique.

The most obvious pitfall associated with this study is the relatively small sample size, which precluded us from probing the relationship between cartilage matrix changes and meniscal status. It has been argued that even minor meniscal injuries could be considered a risk factor of OA [28], which would require a correlation analysis to evaluate its potential impact in the current study. Another major flaw in our study design is the lack of longitudinal follow-up. It would be highly desirable to follow up the patients in different time intervals and compare the differences longitudinally. Further research is needed to determine the utility of T2- and T2^*∗*^-mapping for ACL rupture patients.

In conclusion, our results revealed that the early degeneration can take place within cartilage matrix after acute ACL rupture indicated by different increased T2 and T2^*∗*^ values in various cartilage compartments of the affected knee, especially in the superficial layer of LT. Moreover, A good correlation between reciprocals of T2^*∗*^ and T2 values was observed, and T2^*∗*^-mapping was shown to be more sensitive in detecting cartilage matrix changes that occurred in deep layers than T2-mapping. T2^*∗*^-mapping was also shown to confer the advantages of faster imaging and greater spatial resolution. These results lent support to the employment of T2^*∗*^-mapping as a useful imaging tool for early detection of OA after acute ACL rupture.

## Figures and Tables

**Table 1:** Demographic data of participants in two groups.

Demographics	ACL rupture group	Healthy control group	*P* value
Number, *n*	23	23	/
Injury duration, mo	2–6 (mean, 3.5)	/	/
Interval between injury and surgery, mo	1–4 (mean, 2.5)	/	/
left/right knee	12/11	13/10	0.767
Sex, male/female, *n*	5/18	7/16	0.502
Age, mean ± SD, y	31.5 ± 9.6	29.4 ± 8.4	0.527
BMI, mean ± SD, kg/m^2^	23.4 ± 2.5	23.7 ± 2.2	0.757

ACL, anterior cruciate ligament; BMI, body mass index; SD, standard deviation.

**Table 2:** The T2 and T2^*∗*^ values of cartilage compartments in ACL rupture group and control group.

	T2 value			T2^*∗*^ value		
	ACL rupture	control	*P* value	ACL rupture	control	*P* value
fMF	44.01 ± 5.79	40.39 ± 6.10	0.045	25.32 ± 2.34	23.79 ± 2.60	0.042
sMF	52.37 ± 9.58	46.65 ± 7.64	0.031	27.48 ± 2.46	25.92 ± 2.64	0.043
dMF	33.96 ± 3.66	32.00 ± 5.32	0.154	22.73 ± 2.81	21.86 ± 3.30	0.34
fLF	44.66 ± 7.09	40.44 ± 4.90	0.024	24.37 ± 3.09	22.67 ± 2.16	0.037
sLF	50.84 ± 8.06	45.20 ± 5.63	0.009	26.66 ± 2.99	24.87 ± 1.99	0.022
dLF	38.04 ± 5.82	35.47 ± 4.30	0.096	22.49 ± 3.24	20.53 ± 3.12	0.042
fMT	39.10 ± 9.16	34.27 ± 6.74	0.048	19.17 ± 3.12	17.61 ± 1.88	0.048
sMT	46.32 ± 11.54	40.06 ± 7.67	0.037	21.78 ± 2.40	20.33 ± 2.14	0.039
dMT	26.82 ± 5.35	24.33 ± 6.57	0.165	15.60 ± 2.73	14.52 ± 1.41	0.101
fLT	38.28 ± 6.39	32.43 ± 5.41	0.002	21.27 ± 1.79	19.25 ± 1.28	<0.001
sLT	44.95 ± 6.76	37.23 ± 5.43	<0.001	25.37 ± 1.91	21.59 ± 1.71	<0.001
dLT	26.12 ± 5.24	23.11 ± 5.27	0.058	17.00 ± 1.80	15.00 ± 1.40	<0.001
Pa	36.92 ± 4.70	35.30 ± 5.66	0.298	25.21 ± 1.72	25.31 ± 2.40	0.887
Tr	43.04 ± 3.49	41.43 ± 4.92	0.208	27.03 ± 2.22	27.76 ± 2.14	0.262

ACL, anterior cruciate ligament; fMF, full thickness of medial femur; sMF, superficial medial femur; dMF, deep medial femur; fLF, full thickness of lateral femur; sLF, superficial lateral femur; dLF, deep lateral femur; fMT, full thickness of medial tibia; sMT, superficial medial tibia; dMT, deep medial tibia; fLT, full thickness of lateral tibia; sLT, superficial lateral tibia; dLT, deep lateral tibia; Pa, patella; Tr, trochlea.
